# Supplementary material for: Levels of SARS-CoV-2 antibodies among fully vaccinated individuals with Delta or Omicron variant breakthrough infections
Source: Nat Commun. 2022 Aug 1;13:4466. doi: 10.1038/s41467-022-32254-8 (PMC9342834; doi:10.1038/s41467-022-32254-8)
Supplement: Supplementary file 1 — Supplementary Information [file 41467_2022_32254_MOESM1_ESM.pdf]

Levels of SARS-CoV-2 antibodies among fully vaccinated individuals with Delta or Omicron variant breakthrough infections

Supplementary note 1. Delta variant occurrence in Denmark.

Information regarding the development of Alpha and Delta SARS-CoV-2 variants in Denmark during the study period may be found using the following link https://files.ssi.dk/covid19/virusvarianter/status/virusvarianter-covid19-04082021-ml43 for the report "Status for udvikling af SARS-CoV-2 Varianter der overvåges i Danmark" ("Status of the development of SARS-CoV-2 variants under surveillance in Denmark") of the 4th of August 2021.

Supplementary note 2. Omicron occurrence in Denmark.

Number of confirmed SARS-CoV-2 cases, Omicron cases, samples tested with variant PCR, percentage with variant PCR and percentage of Omicron cases during the study period may be found through the following link:
https://files.ssi.dk/covid19/omikron/statusrapport/rapport-omikronvarianten-07012022-27nk
Statens Serum Institut, "Rapport om Omikronvarianten" ("Report about the omicron variant") 7. January 2022.

Supplementary table 1. Variant classification of breakthrough infections

	**Variant sequence confirmed**		**Total**
	Yes, N (%)	No, N (%)	N
Alpha	3 (75.0)	1 (25.0)	4
Delta	120 (94.5)	7 (5.5)	127
Omicron	40 (11.0)	324 (89.0)	364
Unknown	0	9 (100)	9
Total	163 (32.3)	341 (67.7)	504

Supplementary table 2. Reasons for exclusion.

Total included	*Reason for exclusion*
6950	All participants
6927	Consent withdrawn and requested data deleted
6926	Aged under 18
6925	No vaccine data registered
6918	Non-standard vaccine regimen
6910	Only received 1 dose of vaccine
6525	Previously infected with SARS-CoV-2
6421	No-follow-up (withdrew before baseline)
6076	No follow-up antibody measurements

Supplementary figure 1. Time from baseline to breakthrough infection after initial vaccination by viral variant shown as Kaplan Meier plots. All infections analysis n=6076, Delta infections analysis n=6063, Omicron analysis n=5050

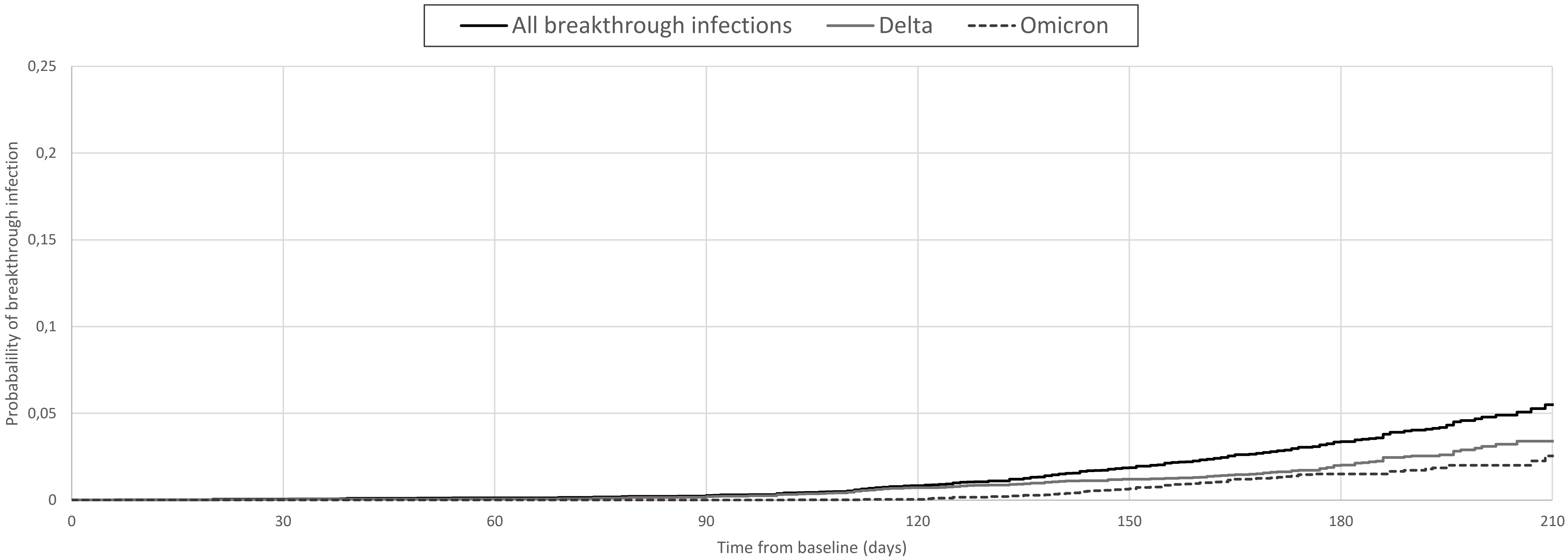

Time		0	30	60	90	120	150	180	210
All	Failed	0	3	8	16	50	110	171	204
	At risk	6076	6071	6053	6036	5975	5135	2882	319
Delta	Failed	0	2	4	12	43	70	100	123
	At risk	6063	6061	6049	6033	5861	5054	5811	302
Omicron	Failed	0	0	0	0	2	31	60	70
	At risk	5050	5050	5050	5046	5019	4257	2127	279

Supplementary figure 2. Time from baseline to breakthrough infections after booster vaccination shown as Kaplan Meier plots. All infections analysis (n=3939), Delta infections analysis (n=1511), Omicron analysis (n=3932)

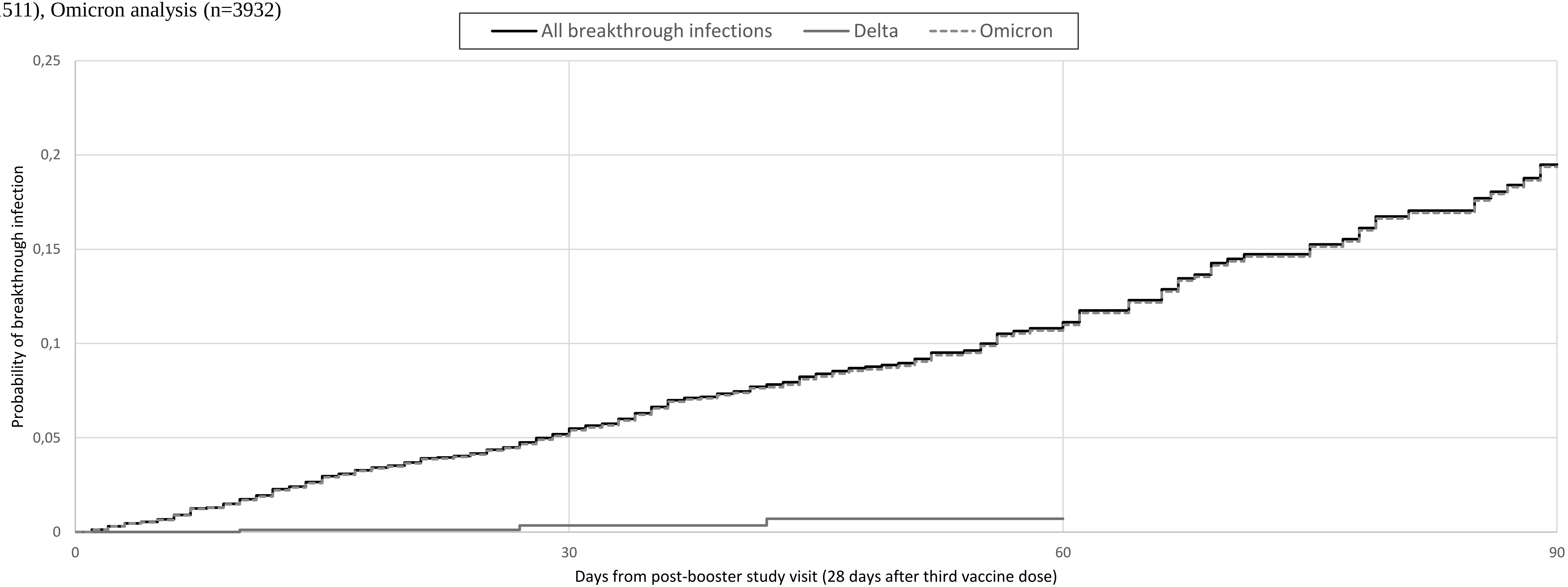

Time		0	30	60	90
All	Failed	0	171	245	279
	At risk	3939	1853	569	214
Delta	Failed	0	2	3	-
	At risk	1511	344	129	-
Omicron	Failed	0	168	241	275
	At risk	3932	1850	568	214

Supplementary figure 3: Incidence rate ratios (IRR) and adjusted incidence rate ratios (aIRR) for breakthrough infections. Calculated using a Poisson regression analysis for each quintile of SARS-CoV-2 anti-receptor binding domain (RBD) IgG log_{10} BAU, stratified by viral variant. Participants were censored at the time of third SARS-CoV-2 vaccination and re-entered the analysis at the time of post-booster visit.

The multivariable models were adjusted for age at enrolment (per year later), gender (male vs female), healthcare worker (no vs yes) and transmission level with two-sided chi-squared tests for each variable in the model. The multivariable model for the Omicron variant did not include transmission level as all Omicron breakthrough infections occurred during the very high transmission period. All variants analysis (n=6076), Delta analysis (n=6063), Omicron (n=5050). The data presented are the aIRR and 95% confidence intervals from the multivariable models.

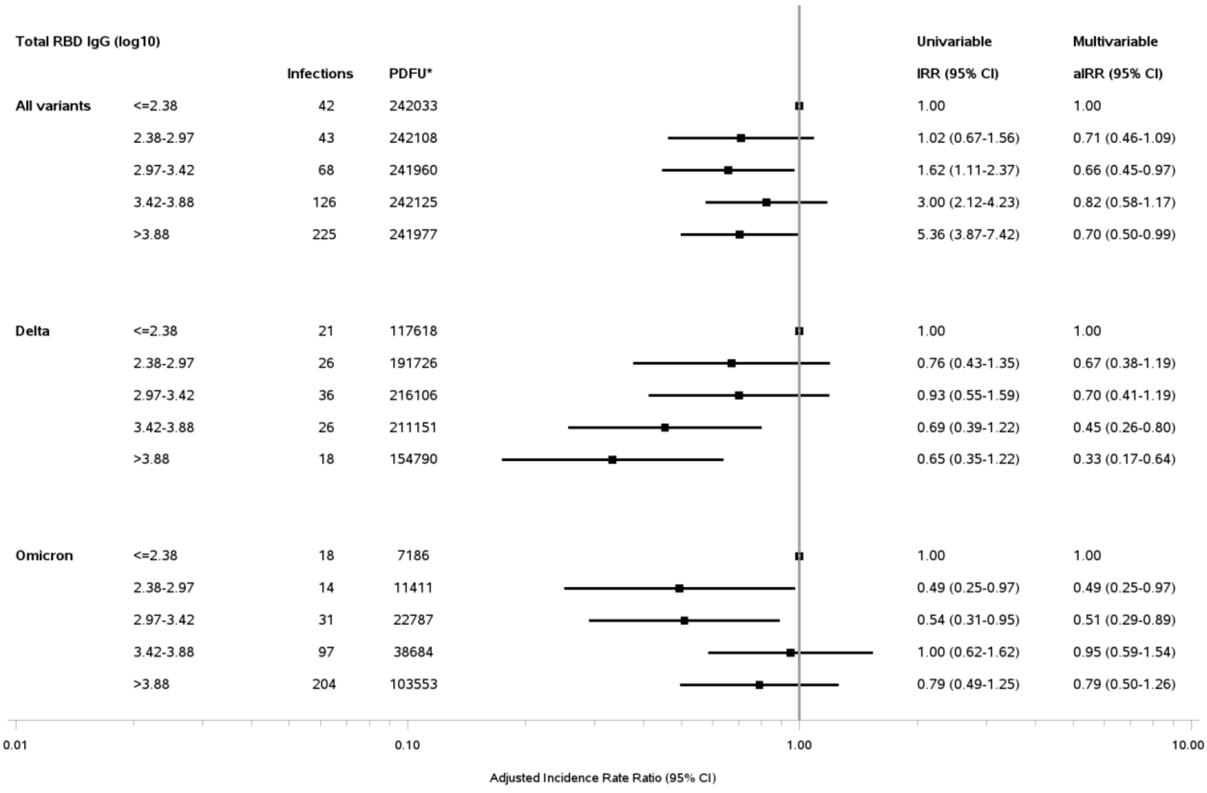

Supplementary figure 4:

Sensitivity analysis forest plot of the adjusted incidence rate ratios (aIRR) for breakthrough infections, using total anti-spike antibody levels in quintiles specific for each variant. Incidence rate ratios (IRR) and adjusted incidence rate ratios (aIRR) for breakthrough infections calculated using a Poisson regression analysis for each quintile of SARS-CoV-2 anti-spike IgG log10 BAU specific for each viral variant. Participants were censored at the time of third SARS-CoV-2 vaccination and re-entered the analysis at the time of post-booster visit. The multivariable models were adjusted for age at enrolment (per year later), gender (male vs female), healthcare worker (no vs yes) and transmission level with two-sided chi-squared tests for each variable in the model. The multivariable model for the Omicron variant did not include transmission level as all Omicron breakthrough infections occurred during the very high transmission period. Delta analysis (n=6063), Omicron (n=5050). The data presented are the aIRR and 95% confidence intervals from the multivariable models.

*Person days of follow-up

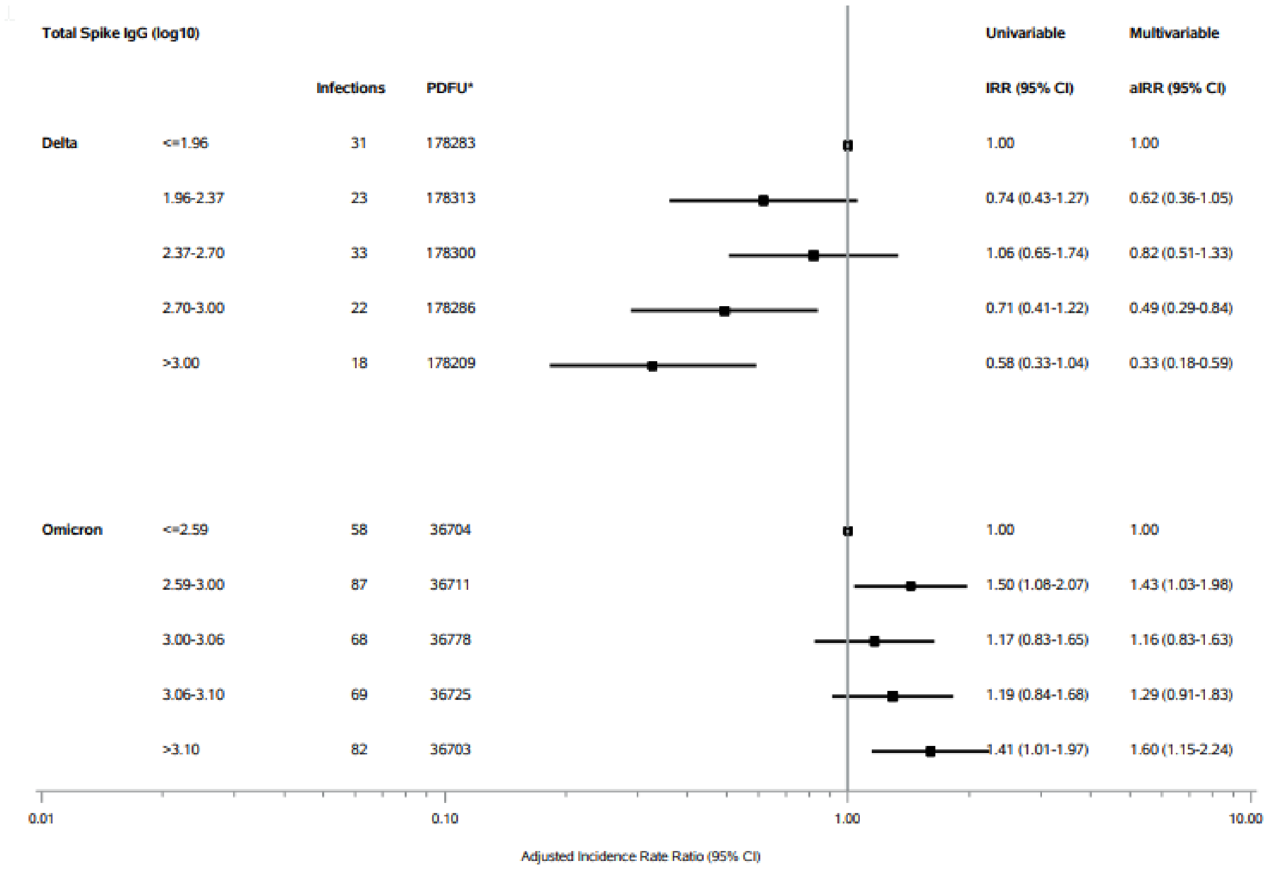

The ENFORCE study group by affiliation.

Coordinating center, Aarhus University/Aarhus University Hospital:

Lars Østergaard, professor
Ole Søgaard, professor
Martin Tolstrup, professor
Katrine Lykkegaard
Vibeke Klastrup, MD
Fredrikke Larsen, MD

Sponsor and Data center, Center of Excellence for Health, Immunity and Infections (CHIP):

Jens Lundgren, professor
Dorthe Raben, MSc
Anna Gro Eilersen
Anna Traytel, MSc
Charlotte Matthews, RN
Christian Møller Jensen
Dennis Kristensen BSc Eng
Erik V. Hansen MSc Eng
Esther Gravholt
Frederik Esmann
Joanne Reekie, PhD
Marie Louise Jakobsen, RN
Nick Normand
Signe Wolsgård Krøyer
Stine F. Jakobsen, MA
Tomas Østergaard Jensen, MD

The Capital Region of Denmark:

Thomas Benfield, professor
Kasper Karmark Iversen, professor
Charlotte Kastberg Levin, RN
Dorthe Petersen, RN
Kamille Fogh, MD
Louise Krohn-Dehli, RN

The Central Denmark Region:

Nina Breinholt Stærke, MD
Ane Søndergaard, RN
Malene Østergaard, RN
Sandra Schieber, RN
Helene Høgh Sørensen
Amalie Glavind Duus
Yordanos Yehdego, RN

The North Denmark Region:

Henrik Nielsen, professor
Jacob Bodilsen, PhD
Kristine Toft Petersen, RN
Maria Ruwald Juhl, RN
Rikke Krog Thisted, RN

The Region of Southern Denmark:

Isik Somuncu Johansen, professor
Christiane Abildgaard
Inge Kristine Holden, PhD
Lone Wulff Madsen, MD
Lykke Larsen, MD
Susan Olaf Lindvig, RN

Region Zealand:

Lothar Wiese, PhD
Janne Lykkegaard Meyerhoff, RN
Lea Guldberg Sander, RN
Lene Surland Knudsen, PhD
Mette Iversen, MD
Sidsel Fjordbak Caspersen, RN

Research Lab, Department of Infectious Diseases, Aarhus University Hospital:

Anna Karina Juhl, PhD
Astrid Korning Hvidt, Cand.Scient.
Eva Baerends, Cand.Scient.
Lisa Loksø Dietz, Cand.Scient.
Mariane Høgsbjerg Schleimann, PhD
Rikke Olesen, PhD
Sidsel Dahl Andersen, PhD
Signe Rode Andreasen, PhD

Statens Serum Institut, serology lab and National Danish Biobank:

Charlotte Sværke Jørgensen, PhD
Karina Meden Sørensen, PhD
Bart Wilkowski, PhD
Jacob Melbye

Scientific Steering Committee:

Bolette Søborg, PhD, Danish Health Authority
Christian Erikstrup, professor, Aarhus University Hospital
Hanne Lomholt Larsen, Danish Medicines Agency
Nanna Aaby Kruse, Danish Medicines Agency
Palle Valentiner-Branth, PhD, Statens Serum Institut
Sisse Ostrowski, professor, Rigshospitalet
Thea Kølsen Fischer, professor, University of Copenhagen
Tyra Grove Krause, PhD, Statens Serum Institut

Other contributors:

David Hougaard, D.Sc, Statens Serum Institut
Erik Jylling, director, Danske Regioner
Anne-Mette Hvass, Professor, Aarhus University Hospital
