# Supplementary material for: Levels of SARS-CoV-2 antibodies among fully vaccinated individuals with Delta or Omicron variant breakthrough infections
Source: Nat Commun. 2022 Aug 1;13:4466. doi: 10.1038/s41467-022-32254-8 (PMC9342834; doi:10.1038/s41467-022-32254-8)
Supplement: Supplementary file 3 — Reporting Summary [file 41467_2022_32254_MOESM3_ESM.pdf]

nature research

Corresponding author(s): Nina Breinholt Stærke

Last updated by author(s): Jul 13, 2022

Reporting Summary

Nature Research wishes to improve the reproducibility of the work that we publish. This form provides structure for consistency and transparency in reporting. For further information on Nature Research policies, see our Editorial Policies and the Editorial Policy Checklist.

Statistics

For all statistical analyses, confirm that the following items are present in the figure legend, table legend, main text, or Methods section.

n/a	Confirmed	
☐	☒	The exact sample size (n) for each experimental group/condition, given as a discrete number and unit of measurement
☐	☒	A statement on whether measurements were taken from distinct samples or whether the same sample was measured repeatedly
☐	☒	The statistical test(s) used AND whether they are one- or two-sided<br>*Only common tests should be described solely by name; describe more complex techniques in the Methods section.*
☐	☒	A description of all covariates tested
☐	☒	A description of any assumptions or corrections, such as tests of normality and adjustment for multiple comparisons
☐	☒	A full description of the statistical parameters including central tendency (e.g. means) or other basic estimates (e.g. regression coefficient) AND variation (e.g. standard deviation) or associated estimates of uncertainty (e.g. confidence intervals)
☐	☒	For null hypothesis testing, the test statistic (e.g. F, t, r) with confidence intervals, effect sizes, degrees of freedom and P value noted<br>*Give P values as exact values whenever suitable.*
☒	☐	For Bayesian analysis, information on the choice of priors and Markov chain Monte Carlo settings
☒	☐	For hierarchical and complex designs, identification of the appropriate level for tests and full reporting of outcomes
☒	☐	Estimates of effect sizes (e.g. Cohen's d, Pearson's r), indicating how they were calculated

Our web collection on statistics for biologists contains articles on many of the points above.

Software and code

Policy information about availability of computer code

Data collection	Data was collected using the REDCap electronic data capture tool (version 10.6.18) hosted at the Capital Region of Denmark.
Data analysis	Data analysis was performed using SAS studio 3.8 on SAS 9.4.

For manuscripts utilizing custom algorithms or software that are central to the research but not yet described in published literature, software must be made available to editors and reviewers. We strongly encourage code deposition in a community repository (e.g. GitHub). See the Nature Research guidelines for submitting code & software for further information.

Data

Policy information about availability of data

All manuscripts must include a data availability statement. This statement should provide the following information, where applicable:

- Accession codes, unique identifiers, or web links for publicly available datasets
- A list of figures that have associated raw data
- A description of any restrictions on data availability

The ENFORCE study is still ongoing. While the study is ongoing data and coding details may be made available to scientists only upon approval of an application sent to the ENFORCE Scientific Steering Committee and approval by relevant authorities. Applications for data must be sent to enforce.rigshospitalet@regionh.dk and will be handled within 6 weeks. Detailed information about data access may be found here: https://chip.dk/Research/Studies/ENFORCE/Study-Governance. Public study reports are available at https://chip.dk/Research/Studies/ENFORCE/Study-Reports.

Field-specific reporting

Please select the one below that is the best fit for your research. If you are not sure, read the appropriate sections before making your selection.

☒ Life sciences ☐ Behavioural & social sciences ☐ Ecological, evolutionary & environmental sciences

For a reference copy of the document with all sections, see nature.com/documents/nr-reporting-summary-flat.pdf

Life sciences study design

All studies must disclose on these points even when the disclosure is negative.

Sample size	Sample size computations are based on simulating 10,000 datasets to compare two vaccines. For a study comparing two vaccines with a sample size of 2,500 per group and with 90% achieving the minimal protective neutralising antibody titre (the chosen measure of vaccine efficacy), it will be possible with 90% certainty to achieve equivalence, with an equivalence margin of δ= +/-2.79%. With a sample size of 2,500 per group our simulations show that with an equivalence margin set at δ=+/-5% we will have sufficient power to ascertain equivalence for all titer levels (40%-90%) with ≥90% certainty.
Data exclusions	For the present study participants with a positive SARS-CoV-2 PCR test anytime prior to 14 days after the second dose of SARS-CoV-2 vaccine were excluded. This was done as the aim was to describe breakthrough infections in fully vaccinated individuals without prior infection. Additionally, participants without any follow-up visits recorded after baseline were excluded.
Replication	This is a observational cohort study measuring antibody levels in participants prior to and following SARS-CoV-2 vaccination. The timing of sampling and methods used to measure antibody levels are described in detail in the protocol and the manuscript, so that it may be reproduced by others.
Randomization	There was no randomization in this study, as it was performed as an observational cohort study.
Blinding	As the study was observational, there were no blinding.

Reporting for specific materials, systems and methods

We require information from authors about some types of materials, experimental systems and methods used in many studies. Here, indicate whether each material, system or method listed is relevant to your study. If you are not sure if a list item applies to your research, read the appropriate section before selecting a response.

Materials & experimental systems

n/a	Involved in the study	
☐	☒	Antibodies
☒	☐	Eukaryotic cell lines
☒	☐	Palaeontology and archaeology
☒	☐	Animals and other organisms
☐	☒	Human research participants
☐	☒	Clinical data
☒	☐	Dual use research of concern

Methods

n/a	Involved in the study	
☒	☐	ChIP-seq
☒	☐	Flow cytometry
☒	☐	MRI-based neuroimaging

Antibodies

Antibodies used	*Describe all antibodies used in the study; as applicable, provide supplier name, catalog number, clone name, and lot number.*
Validation	*Describe the validation of each primary antibody for the species and application, noting any validation statements on the manufacturer's website, relevant citations, antibody profiles in online databases, or data provided in the manuscript.*

Human research participants

Policy information about studies involving human research participants

Population characteristics	Participants in this study were Danish adult citizens receiving a SARS-CoV-2 vaccine through the Danish National SARS-CoV-2 vaccination program. All participants are 18 years or older. There were no selection based on sex or comorbidities. The study does not involve genotypic information.
Recruitment	All citizens that had booked an appointment to be vaccinated through the Danish National SARS-CoV-2 program at selected vaccine centers received an invitation through electronic mailing and/or posters at the vaccination clinic. Vaccine groups differed regarding comorbidities and age, due to the prioritization of specific vaccines to specific groups in the vaccination

program, and due to limited supply of vaccines at different time points. The present study does not compare vaccine groups, but antibody levels at breakthrough infections with two different SARS-CoV-2 variants. There may have been some selection bias in the studied population, as some citizens that were offered vaccination did not choose to participate. However, the present study investigates the difference between groups infected with two different viral variants, and it is not likely that there is any association between participation in the study and infection with either of the viral variants, and hence the bias does not affect the results.

Ethics oversight

The Regional Ethics committe of Central Denmark approved this study.

Note that full information on the approval of the study protocol must also be provided in the manuscript.

Clinical data

Policy information about clinical studies

All manuscripts should comply with the ICMJE guidelines for publication of clinical research and a completed CONSORT checklist must be included with all submissions.

Clinical trial registration

Clinicaltrials.gov identifier: NCT04760132

Study protocol

https://chip.dk/Research/Studies/ENFORCE/Study-Documents

Data collection

Data collection was performed at 7 sites (Aalborg, Silkeborg, Aarhus, Odense, Roskilde, Hvidovre and Herlev) spread across the 5 Danish regions. Each site recruited participants from regional vaccination centers. Recruitment was performed from february to july 2021. Data collection started in february 2021 and is still ongoing. Participants will be followed for a total of 48 months.

Outcomes

At the time the study protocol was drafted, it was believed that there would be a “sero-protective level of antibodies” that would protect against COVID-19 similarly to what the sero-protective titer for Hep B and invasive pneumococcal disease. Therefore the primary endpoint was minimal protective neutralizing antibody titer. We now know that there is no minimal level of neutralizing antibodies sufficient to protect a vaccinated individual from becoming infected with SARS-CoV-2. Thus, in the present manuscript, we report the modified primary endpoint – level of SARS-CoV-2 spike antibody associated risk of breakthrough infection. This was assessed by comparing the levels of antibodies at time of breakthrough infection stratified by viral variant, as described in the manuscript.
Secondary outcomes were: detailed immunological assessment in subgroups of participants of markers of cellular immunity, breakthrough infections by vaccine type hroughout the 24-month follow-up period, local and systemic vaccine reactions within 14 days of vaccination by vaccine type, grade 3 and 4 adverse event and serious adverse events by vaccinet ype and grade 1 and 2 adverse events by vaccine type. Secondary outcomes were not yet assessed as the study is still ongoing.
